# Neovascular Niche for Human Myeloma Cells in Immunodeficient Mouse Bone

**DOI:** 10.1371/journal.pone.0030557

**Published:** 2012-02-07

**Authors:** Hirono Iriuchishima, Keiyo Takubo, Yoshitaka Miyakawa, Ayako Nakamura-Ishizu, Yoshiteru Miyauchi, Nobuyuki Fujita, Kana Miyamoto, Takeshi Miyamoto, Eiji Ikeda, Masahiro Kizaki, Yoshihisa Nojima, Toshio Suda

**Affiliations:** 1 Department of Cell Differentiation, The Sakaguchi Laboratory of Developmental Biology, Keio University School of Medicine, Tokyo, Japan; 2 Division of Hematology, Department of Internal Medicine, Keio University School of Medicine, Tokyo, Japan; 3 Department of Orthopedic Surgery, Keio University School of Medicine, Tokyo, Japan; 4 Department of Pathology, Yamaguchi University Graduate School of Medicine, Ube, Yamaguchi, Japan; 5 Department of Hematology, Saitama Medical Center, Saitama Medical University, Saitama, Japan; 6 Department of Medicine and Clinical Science, Gunma University Graduate School of Medicine, Maebashi, Gunma, Japan; RWTH Aachen University Medical School, Germany

## Abstract

The interaction with bone marrow (BM) plays a crucial role in pathophysiological features of multiple myeloma (MM), including cell proliferation, chemoresistance, and bone lesion progression. To characterize the MM-BM interactions, we utilized an *in vivo* experimental model for human MM in which a GFP-expressing human MM cell line is transplanted into NOG mice (the NOG-hMM model). Transplanted MM cells preferentially engrafted at the metaphyseal region of the BM endosteum and formed a complex with osteoblasts and osteoclasts. A subpopulation of MM cells expressed VE-cadherin after transplantation and formed endothelial-like structures in the BM. CD138^+^ myeloma cells in the BM were reduced by p53-dependent apoptosis following administration of the nitrogen mustard derivative bendamustine to mice in the NOG-hMM model. Bendamustine maintained the osteoblast lining on the bone surface and protected extracellular matrix structures. Furthermore, bendamustine suppressed the growth of osteoclasts and mesenchymal cells in the NOG-hMM model. Since VE-cadherin^+^ MM cells were chemoresistant, hypoxic, and HIF-2α-positive compared to the VE-cadherin^−^ population, VE-cadherin induction might depend on the oxygenation status. The NOG-hMM model described here is a useful system to analyze the dynamics of MM pathophysiology, interactions of MM cells with other cellular compartments, and the utility of novel anti-MM therapies.

## Introduction

Multiple myeloma (MM) is a clonal disorder of late-stage B cells, in which transformed plasma cells expand and accumulate in the bone marrow (BM), leading to cytopenia, bone resorption and the production of the characteristic monoclonal immunoglobulin [1]. The bone marrow (BM) is believed to play a crucial role in the pathophysiological features of MM, including cell proliferation, chemoresistance and bone lesion progression. The “MM niche” is composed of osteoblasts, osteoclasts, vascular endothelial cells, stromal cells, adipocytes, and extracellular matrix (ECM) proteins [2]. MM cells are thought to be maintained by these various stromal cells [3], which regulate their survival, proliferation and apoptosis. However, the cellular and anatomical features of stromal cells have not been thoroughly examined in the MM. In this study, the MM niche was characterized in the BM of a mouse transplanted with human MM cells.

Recently, chemotherapeutic strategies for the treatment of MM have significantly advanced, including the development of anti-angiogenic drugs [4]. Using the MM mouse model, the suppressive effects of bendamustine, a bifunctional chemotherapeutic agent with a nitrogen mustard structure and a purine benzimidazole ring, were examined. Because of its unique structure, bendamustine exhibits only partial cross-resistance with other alkylators [5]–[7] and is widely used for the treatment of various hematological malignancies, including low-grade lymphoma and MM [8], [9]. Although the clinical utility of bendamustine in the treatment of MM has been clearly demonstrated [10], [11], the specific cellular and molecular effects of bendamustine on MM cells and the MM niche *in vivo* have not been determined. Therefore, this issue was investigated in a mouse model of human MM.

## Results

### Establishment of a human multiple myeloma in NOD/SCID/γ_c_^null^ mice

**Figure 1:**
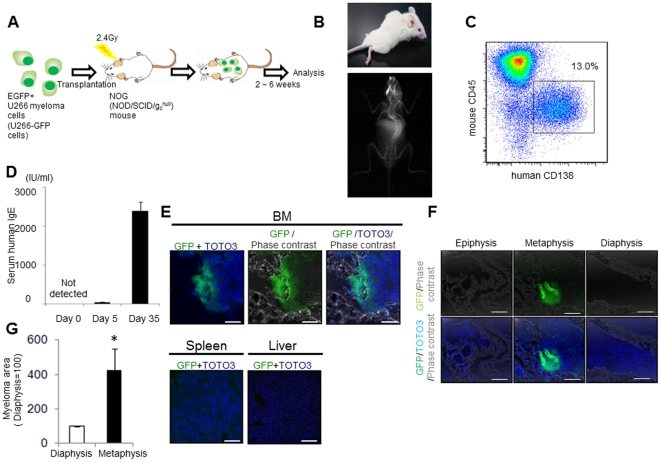
Interaction between multiple myeloma cells and the niche in an *in vivo* human myeloma model. **A.** Study design of the NOG-hMM model. Two million EGFP^+^ U266 myeloma (U266-EGFP) cells were intravenously inoculated into 2.4 Gy irradiated NOG (NOD/SCID/g_c_^null^) mice. Two to six weeks after transplantation, mice were sacrificed for analysis. **B.** Representative clinical manifestation (upper panel) and radiologic examination (lower panel) of mice 4 weeks after transplantation. All mice showed hind leg paralyses (left) associated with spinal compression fractures (left; arrow). This clinical manifestation was observed in more than 20 mice. **C.** Representative FACS plot of U266-EGFP cell-transplanted NOG mice. Bone marrow of NOG mice was analyzed by FACS 4 weeks after U266-EGFP cell transplantation using anti-human CD138 antibody and anti-murine CD45 antibody. The experiment was performed in more than forty mice with similar results. **D.** Serum levels of human IgE. Human IgE levels in the serum of NOG mice at 0 (non-transplanted, non-irradiated mice), 5 or 35 days after transplantation with U266-EGFP cells were analyzed by ELISA (n = 3–5, mean±SD). The experiment was performed twice with similar results. **E.** Double immunohistochemistry (IHC) of EGFP (green) and TOTO3 (blue) of the BM (upper panels), spleen or liver (lower panels) at 2 weeks after transplantation. U266-EGFP cells engrafted only in the BM. Representative data from 10 to 20 fields from the BM of two mice from three independent experiments are shown. Bars = 200 µm. **F.** Immunohistochemical (IHC) analysis of EGFP (green) and TOTO3 (blue) at the anatomical region of long bones at the early stage. Representative data from 10 to 20 fields from the BM of two mice from three independent experiments are shown. Bars = 200 µm. **G.** Quantification of U266-EGFP cells at the diaphysis and the metaphysis of the long bones. U266-EGFP at the metaphysis regions is represented relative to the diaphysis region (diaphysis = 100, n = 3, mean ± SEM). The experiment was performed twice with similar results.

When U266 cells, a human IgE-producing myeloma cell line, were transplanted into super-immunodeficient NOG mice [12], the cells engrafted and proliferated in the BM but not in any other organs. These mice exhibited various MM clinical symptoms, including the development of osteolytic bone lesions [13]. To analyze the myeloma niche more precisely, U266 cells stably expressing EGFP (U266-GFP) were transplanted into NOG mice (NOG-hMM model). 2×10^6^ U266-GFP cells were intravenously inoculated into irradiated NOG mice (2.4 Gy), and recipient mice were sacrificed 2–6 weeks after transplantation to assess the establishment of the MM (Fig. 1A). After 4 weeks, the recipient NOG mice all developed bilateral paralysis and pathological fractures. Radiologic examination revealed severe scoliosis (Fig. 1B). Because GFP fluorescence in transplanted U266-GFP cells tended to decrease *in vivo* (data not shown), we used CD138, a pan-myeloma marker, to detect U266-GFP cells. Flow cytometric analysis of the BM revealed myeloma cells positive for human CD138 and negative for mouse CD45 (Fig. 1C). Furthermore, human IgE was detected in the sera of recipient mice at 5 days after transplantation and was drastically increased at day 35 post-transplantation (Fig. 1D).

### The human MM niche in the BM of NOG mice

Engraftment of GFP-positive myeloma cells was detected in the BM but not in the spleen, liver, or any other organs (Fig. 1E). To characterize the myeloma niche *in vivo*, the recipient long bones, including the femur and tibia, were analyzed because their anatomical and histological structures in the hematopoietic niche have been well characterized. Within the BM of the long bone, myeloma cells were located mainly in the endosteal zone in the femur (Fig. 1E), indicating that the endosteal zone of the BM is the preferential location for myeloma engraftment. Next, the temporal distribution of myeloma cells in the BM was investigated. At the early stage (2–4 weeks after transplantation), myeloma cells were primarily observed at the metaphyseal region (the wide portion of a long bone adjacent to the epiphyseal plate) but not in the epiphyseal region (the rounded end of a long bone) or the diaphyseal region (the mid-section of a long bone) of the BM (Fig. 1F). CD138^+^ myeloma cells at the later stage (4–9 weeks after transplantation) were distributed mainly in the metaphyseal region and were relatively rare in the diaphyseal region in the femur (Fig. 1G).

**Figure 2:**
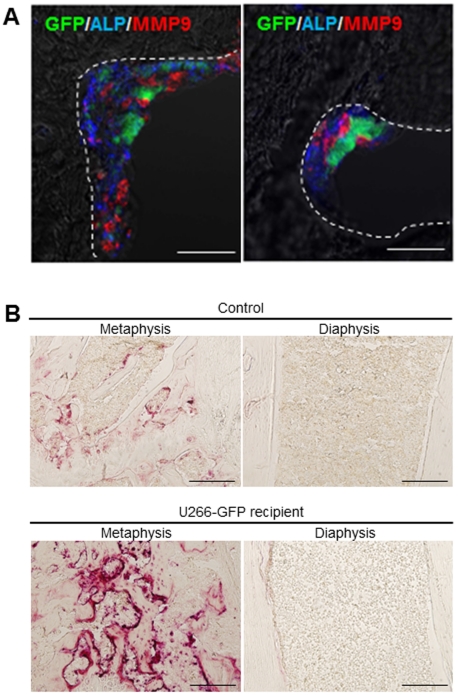
Dynamics of osteoblasts and osteoclasts at the MM niche. **A.** Triple IHC of GFP (green), ALP (blue) and MMP-9 (red) in the metaphyseal region at 2 weeks after U266-EGFP cell transplantation. Myeloma cells form a cellular complex with osteoblasts and osteoclasts *in vivo*. Representative data from 10 to 20 fields from the BM of two mice from three independent experiments are shown. Bars = 50 µm. **B.** Regional differences in TRAP-positive osteoclast cells in the long bones. Micrographs of TRAP staining of the metaphysis and the diaphysis at 4 weeks after U266-EGFP cell transplantation. Whereas the development of TRAP-positive osteoclast cells is barely discernable in the diaphysis region, a number of TRAP-positive osteoclast cells are evident in the metaphysis region. The experiment was performed three times with similar results. Bars = 200 µm.

Immunohistochemical analysis of the MM component at the early stage (2 weeks after transplantation) revealed that GFP-positive myeloma cells formed a complex with alkaline phosphatase (ALP)-positive osteoblasts and matrix metalloprotease (MMP) 9-positive osteoclasts in the femoral endosteum (Fig. 2A). Under normal conditions, NOG mice possess more tartrate-resistant acid phosphatase (TRAP)-positive osteoclasts in the metaphyseal region than in the diaphyseal region in the femur (Fig. 2B, left panels). At 4 weeks after transplantation, increased TRAP activity, which represents an increase in osteoclasts, was observed in the metaphyseal region but not in the femoral diaphyseal region (Fig. 2B, right panels). These data suggest that the myeloma niche is located in the metaphyseal endosteal zone of the longitudinal bone and is composed of osteoblasts and osteoclasts *in vivo*.

### VE-cadherin expression and vascular mimicry of the MM cells

**Figure 3:**
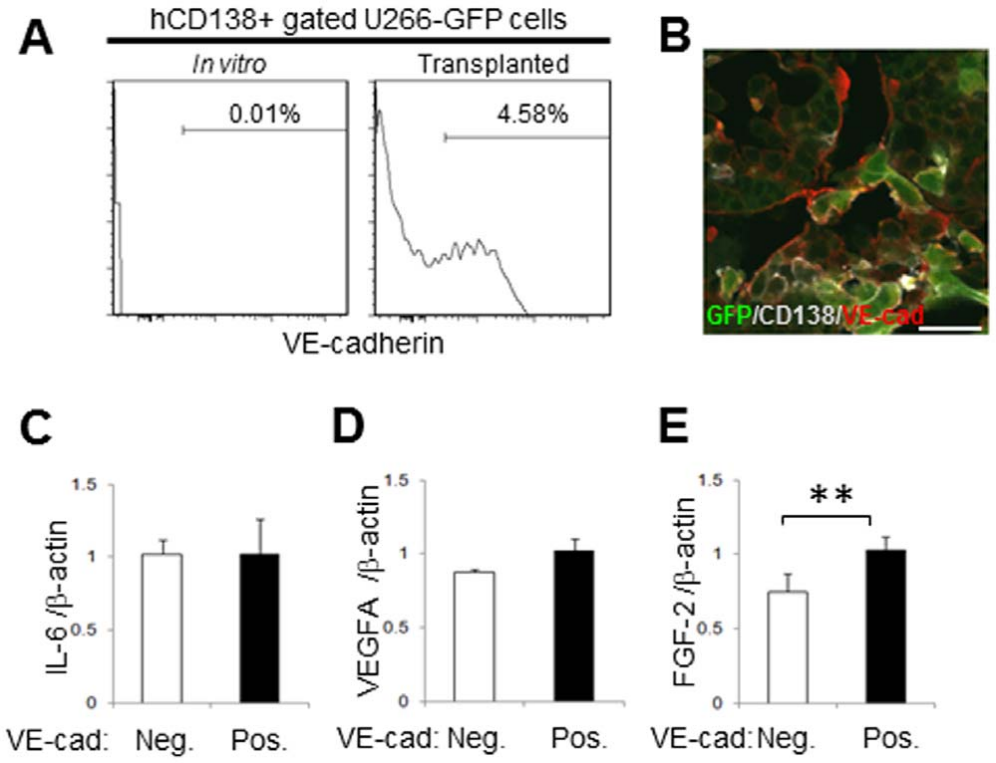
MM cells express VE-cadherin and form endothelial-like structures in the BM. **A.** Flow cytometric detection of VE-cadherin in U266-EGFP cells *in vitro* (left) or transplanted U266-EGFP cells (right) using anti-human VE-cadherin antibody. The experiment was performed with more than twenty mice with similar results. **B.** Triple IHC of GFP (green), human CD138 (gray) and VE-cadherin (red) within the diaphyseal region at 4 weeks after U266-EGFP cell transplantation. Representative data from four fields from the BM of three mice in two independent experiments are shown. Bars = 20 µm. **C–E**. Quantitative PCR analysis of IL-6 (**B**), VEGFA (**C**) or FGF-2 (**D**) transcripts in the VE-cadherin^+^ or VE-cadherin^−^ MM fractions five weeks after U266-EGFP cell transplantation. Each value was normalized to β-actin expression and is expressed as the fold induction compared to VE-cadherin^+^ samples (mean ± SD, **P<0.01, n = 4). The experiment was performed twice with similar results.

It is well known that increased vasculature accompanies active myeloma [14]–[16]. Therefore, the relationship between endothelial and myeloma cells was analyzed. Flow cytometric analysis revealed a VE-cadherin^+^ subpopulation of human CD138^+^ myeloma cells *in vivo* (Fig. 3A). Interestingly, these VE-cadherin^+^ cells were not detected *in vitro* and were only observed after the transplantation of U266-GFP cells into NOG mice (Fig. 3A). These VE-cadherin^+^ cells formed vascular-like structures in the femoral BM (Fig. 3B). To further characterize the VE-cadherin^+^ cell population, VE-cadherin^+^ cells and VE-cadherin^−^ cells were sorted from the CD138^+^ fraction and the expression of angiogenesis-related genes in each subpopulation was analyzed using human-specific primer sets. MM cells have been reported to secrete vascular endothelial growth factor (VEGF)-A into the BM microenvironment to induce angiogenesis [17]. Consistent with previous reports, human interleukin (IL)-6, VEGF-A, and fibroblast growth factor (FGF)-2 were expressed in both fractions (Fig. 3C–E). In particular, the expression of FGF-2 was significantly increased in the VE-cadherin^+^ fraction (Fig. 3E). These data suggest that FGF-2 is involved in myeloma angiogenesis and may induce the formation of vascular endothelial-like structures by a subpopulation of VE-cadherin^+^ cells. Although it is rare, human cancer cells are known to sometimes fuse with endothelial cells to form hybrid cells [18]. To address this possibility, fluorescence *in situ* hybridization (FISH) analysis was performed on the VE-cadherin^+^ and VE-cadherin^−^ CD138^+^ cells from NOG-hMM mice. Cultured U266-GFP cells were used as a negative control. Fused cells were very rare (<0.1%) and not specific to the VE-cadherin^+^ subpopulation (Table S1). These data indicate that human VE-cadherin^+^ CD138^+^ cells were generated in the BM microenvironment *in vivo*.

### *In vivo* effect of bendamustine

**Figure 4:**
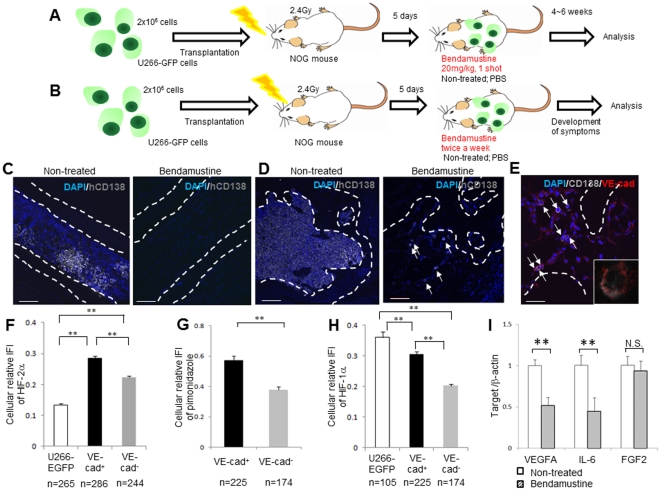
Bendamustine-treated MM cells remaining at the metaphyseal region express VE-cadherin and show hypoxic phenotypes. **A.** A single dose of bendamustine (20 mg/kg) or an equivalent amount of PBS was administered intraperitoneally to engrafted mice 5 days after U266-EGFP cell transplantation. Four to six weeks after administration, mice were sacrificed for analysis. **B.** Over the course of a week, two sequential doses of bendamustine (20 mg/kg) or equivalent amounts of PBS were administered intraperitoneally to engrafted mice 5 days after U266-EGFP cell transplantation. These mice were sacrificed for analysis when the mice had developed disease symptoms. **C, D.** IHC of human CD138 (gray) at the diaphyseal region (**C**) or the metaphyseal region (**D**) at 4 weeks after treatment in bendamustine-treated mice (right panel) or non-treated (PBS-treated) mice (left panel). Representative data from five fields of the BM of two mice per group in two independent experiments are shown. In A and B, the left bars = 200 µm, and in B, the right bar = 100 µm. **E.** Double IHC of human CD138 (gray) and VE-cadherin (red) at 4 weeks after treatment in bendamustine-treated mice. The arrows indicate human CD138-positive MM cells. Representative data from five fields of the BM of two mice in two independent experiments are shown. Bar = 50 µm. **F–H.** Quantification of HIF-2α (**F**) or HIF-1α (**H**) protein levels in cultured U266-EGFP cells, or VE-cadherin^+^ or VE-cadherin^−^ fractions, 6 weeks after U266-EGFP cell transplantation and quantification of pimonidazole retention (**G**) in VE-cadherin^+^ or VE-cadherin^−^ fractions at 6 weeks after transplantation. The experiment was performed twice with similar results. **I.** Human CD138^+^ mouse CD45^−^ MM cells were sorted from non-treated or bendamustine-treated mice, and VEGFA, IL-6 or FGF-2 transcripts were examined by qRT-PCR. Each value was normalized to β-actin expression (mean ± SD, **P<0.01, n = 4). The experiment was performed twice with similar results.

Using the NOG-hMM model, the antitumor effect of bendamustine was assessed *in vivo*. A single dose (Fig. 4A) or two doses over the course of a week (Fig. 4B) of bendamustine (20 mg/kg) were injected intraperitoneally into myeloma-inoculated NOG mice 5 days after transplantation, at which point MM cells had already engrafted into the BM. These mice were analyzed at 4–6 weeks after administration (Fig. 4A) or when the mice had developed symptoms (Fig. 4B). At the early stage after transplantation, myeloma cells were only observed at the metaphyseal region of the BM (Fig. 1F). However, in the later stage (Fig. 4C left), at which time the recipient mice were treated with bendamustine, myeloma cells had infiltrated the diaphyseal zone of the bone. In response to sequential bendamustine administration, femoral diaphyseal myeloma cells were largely depleted (Fig. 4C). In non-treated mice, myeloma cells accumulated in the metaphyseal region rather than in the diaphyseal region of the femur (Fig. 4D, left). Although bendamustine treatment largely depleted the MM cells (Fig. 4D, right), there were some instances of bendamustine-resistant myeloma cells which were positive for VE-cadherin (Fig. 4E).

### Remaining VE-cadherin^+^ MM cells express HIF-2α in response to hypoxia

The hypoxic status of the BM is an important niche characteristic [19]. The hypoxia-inducible transcription factor HIF-2α is known to induce VE-cadherin expression [20]. Therefore, levels of the hypoxic marker pimonidazole and levels of HIF-1α and HIF-2α were compared between the VE-cadherin^+^ and VE-cadherin^−^ MM fractions. The expression level of HIF-2α was higher in the VE-cadherin^+^ cell fraction than in the VE-cadherin^−^ cell fraction, and, interestingly, was induced after transplantation into NOG mice (Fig. 4F). VE-cadherin^+^ MM cells showed higher levels of pimonidazole and HIF-1α protein than VE-cadherin^−^ MM cells (Fig. 4G, H). These data indicate that the HIF-2α-VE-cadherin pathway is activated by hypoxia in a subset of MM cells.

The effect of bendamustine treatment on the paracrine loop of angiogenic factors was also examined *in vivo*. Human VEGFA and IL-6 transcript levels were significantly reduced after bendamustine treatment, but FGF-2 transcript levels remained unchanged (Fig. 4I). These results indicate that bendamustine could suppress myeloma cells and affect the myeloma niche by inhibiting VEGFA and IL-6 production.

### Characterization of the extracellular matrix components and mesenchymal cells in the MM niche

**Figure 5:**
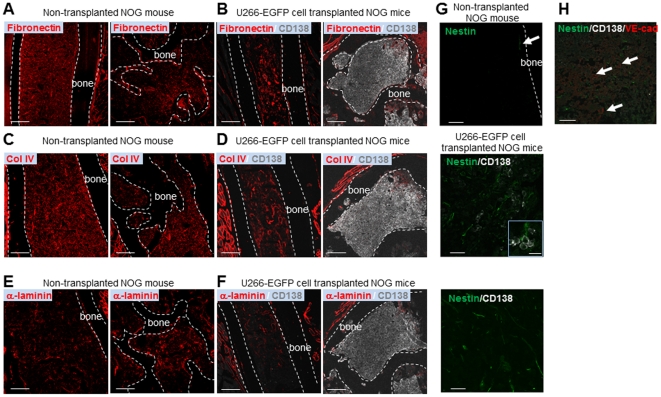
Disorganized ECM components and increased mesenchymal cells in the MM niche. **A, B.** IHC of fibronectin (red; **A**), or fibronectin (red) and human CD138 (gray; **B**), at the diaphyseal region (left) or metaphyseal region (right) of long bones of a non-transplanted NOG mouse (**A**) or U266-EGFP cell-transplanted NOG mice (**B**). Representative data from five fields from the BM of one non-transplanted NOG mouse and five U266-EGFP cell-transplanted mice in two independent experiments are shown. Bars = 200 µm. **C, D.** IHC of type IV collagen (red; **C**), or type IV collagen (red) plus human CD138 (gray; **D**), at the diaphyseal region (left) or metaphyseal region (right) of the long bones of a non-transplanted NOG mouse (**C**) or U266-EGFP cell-transplanted NOG mice (**D**). Representative data from five fields from the BM of one non-transplanted NOG mouse and five U266-EGFP cell-transplanted mice from two independent experiments are shown. Bars = 200 µm. **E, F.** IHC of α-laminin (red; **E**), or α-laminin (red) plus human CD138 (gray; **F**), at the diaphyseal region (left) or metaphyseal region (right) of the long bones of a non-transplanted NOG mouse (**E**) or U266-EGFP cell-transplanted NOG mice (**F**). Representative data from five fields from the BM of one non-transplanted NOG mouse and five U266-EGFP cell-transplanted mice from two independent experiments are shown. Bars = 200 µm. **G.** IHC of nestin (green; upper panel), or nestin (green) plus human CD138 (gray; middle and lower panels), of the long bones of a non-transplanted NOG mouse (upper panel) or U266-EGFP cell-transplanted NOG mice (middle and lower panels). Nestin^+^ mesenchymal cells were rare in normal NOG BM (upper panel; arrow) but increased in U266-EGFP cell-transplanted mice independent of vascular association (lower two panels). Representative images from five fields from the BM of one non-transplanted NOG mouse and five U266-EGFP cell-transplanted mice from two independent experiments are shown. Bars = 40 µm. (Magnified figure bar = 20 µm.) **H.** Triple IHC of nestin (green), CD138 (gray) and VE-cadherin (red) within the metaphyseal regions at 4 weeks after U266-EGFP cell transplantation. Arrows indicate vessels. Representative data from 10 to 20 fields from the BM of two mice from three independent experiments are shown. Bar = 40 µm.

To further characterize the myeloma niche, the expression and structure of the extracellular matrix (ECM) components of the BM, including fibronectin, type IV collagen (col IV) and α-laminin, were analyzed. Metaphyseal MM BM of the femur showed disruption of each of these ECM components (Fig. 5B, right; 5D, right; 5F, right). In addition, ECM structures in the non-MM diaphyseal areas of the femur were increased and disorganized compared to normal NOG mice (Fig. 5A–F). The presence of another component of the BM, nestin^+^ mesenchymal cells [21], was also examined in myeloma. While nestin^+^ cells were rare in the femoral BM of normal NOG mice (Fig. 5G, top), nestin^+^ cells were drastically increased in the femoral BM of MM mice (Fig. 5G, middle). Interestingly, CD138^+^ MM cells were located in close contact with nestin^+^ cells (Fig. 5G, middle). Moreover, nestin^+^ cells in NOG-hMM mice were increased in the non-MM areas of the femoral BM (Fig. 5G, bottom). Although nestin^+^ cells are normally located adjacent to the vasculature [21], nestin^+^ cells in NOG-hMM mice did not co-localize with endothelial cells (Fig. 5H). These data suggest that myeloma cells promote ECM reorganization and nestin^+^ cell proliferation, which might create an abnormal microenvironment that promotes tumor progression and drug resistance in MM.

### Bendamustine maintains the normal BM niche and suppresses pathological myelofibrosis

**Figure 6:**
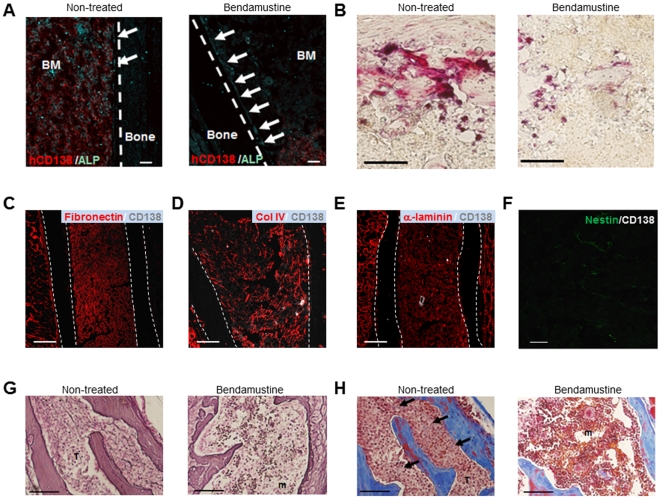
Bendamustine maintains the normal BM niche *in vivo*. **A.** Double IHC of human CD138 (red) and ALP (cyan) at 4 weeks after treatment in bendamustine-treated mice (right panel) or non-treated mice (left panel). The arrows indicate the osteoblast lining on the bone surface. Representative data from seven fields of the BM of two mice per group from two independent experiments are shown. Bars = 60 µm. **B.** Representative photomicrographs of TRAP staining of the metaphyseal region of the femur at 4 weeks after treatment in bendamustine-treated mice (right panel) or non-treated mice (left panel). Representative data from three fields from the BM of four non-treated mice or two bendamustine-treated mice from two independent experiments are shown. Bars = 100 µm. **C–E.** Double IHC of fibronectin (red; **C**), type IV collagen (**D**) or α-laminin (**E**) with human CD138 (gray) at 4 weeks after bendamustine treatment. Representative data from five fields of the BM of two bendamustine-treated mice from two independent experiments are shown. Bars = 200 µm. **F.** Double IHC of nestin (green) and human CD138 (gray) at 4 weeks after bendamustine treatment. Representative data from five fields of the BM of two bendamustine-treated mice from two independent experiments are shown. Bar = 40 µm. **G.** Histological studies of the thoracic vertebrae of NOG mice 4 weeks after treatment in bendamustine-treated mice (right panel) or non-treated mice (no treatment; left panel). Reticulin staining: T, tumor; m, marrow. Representative data from five fields of the BM of five mice per group from two independent experiments are shown.Bars = 100 µm. **H.** Histological studies of the thoracic vertebrae of NOG mice 4 weeks after treatment in bendamustine-treated mice (right panel) or non-treated mice (left panel). Masson trichrome staining: T, tumor; m, marrow. Representative data from five fields of the BM of five mice per group from two independent experiments are shown. Bars = 100 µm.

After a single bendamustine treatment, the osteoblast lining on the femoral surface of the treated mice, which was disrupted without treatment, was observed to be intact (Fig. 6A). TRAP staining of single bendamustine-treated mice showed a decrease in the number and size of osteoclasts compared to PBS-treated (non-treated) mice (Fig. 6B), while bendamustine had no direct effect on osteoclast numbers (data not shown). ECM structures of the femoral BM, such as fibronectin, col IV and α-laminin, were maintained by single bendamustine treatment (Fig. 6C–E). Moreover, treatment with bendamustine also suppressed the proliferation of nestin^+^ cells (Fig. 6F). These data suggest that bendamustine treatment affects not only myeloma cells but also the surrounding niche cells.

Because myeloma often includes pathological myelofibrosis [22], the effect of bendamustine on myelofibrosis was examined. Moderate reticulin-positive fibrosis was noted in the non-treated BM of thoracic vertebrae (Fig. 6G, left); however, no fibrosis was observed in the BM of mice treated with a single dose of bendamustine (Fig. 6G, right). Masson trichrome staining, which detects collagen deposition, revealed several significant fibrotic regions in the non-treated BM of thoracic vertebrae (Fig. 6H, left). On the other hand, no collagen fibrosis was evident in the BM of mice treated with a single dose of bendamustine (Fig. 6H, right). These data indicate that bendamustine suppresses pathological myelofibrosis *in vivo*.

### Hematological maintenance by bendamustine administration

**Figure 7:**
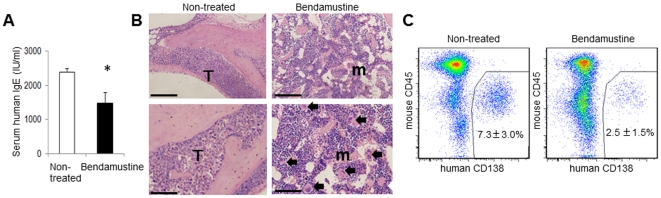
Bendamustine suppresses MM cells *in vivo*. **A.** Serum levels of human IgE. Human IgE levels in the serum of NOG mice 4 weeks after treatment with bendamustine were analyzed by ELISA. Non-treated mice were injected with PBS (n = 5, mean ± SD). The experiment was performed three times with similar results. **B.** Histological studies of the thoracic vertebrae of NOG mice 4 weeks after treatment with bendamustine (right panel) or non-treatment (left panel). Massive infiltration of U266-EGFP cells was observed in non-treated mice. However, an overall reduction of U266-EGFP cells and maintenance of normal hematopoiesis were observed in bendamustine-treated mice. HE staining: T, tumor; m, marrow; arrow, megakaryocyte. Representative data from five fields from the BM of five mice per group from two independent experiments are shown. Upper panel bars = 200 µm. Lower panel bars = 100 µm. **C.** FACS analysis of U266-EGFP cells at 4 weeks after treatment in bendamustine-treated or non-treated mice (n = 5, mean ± SD). Representative FACS plots of at least 10 mice from two independent experiments are shown.

Human IgE levels in the serum of mice receiving a single bendamustine administration were significantly suppressed compared to the non-treated group (Fig. 7A). In histological studies, massive infiltration of myeloma cells was observed in the thoracic vertebrae of non-treated mice, and normal hematopoiesis was minimal (Fig. 7B, left). However, a number of normal hematopoietic cells, including megakaryocytes, were observed in the BM of single bendamustine-administrated NOG mice (Fig. 7B, right). Flow cytometric analysis of non-treated and bendamustine-treated groups showed that the percentage of myeloma cells was decreased in bendamustine-treated mice (Fig. 7C).

### Bendamustine induces p53-dependent apoptosis and suppresses myeloma cells *in vivo*

**Figure 8:**
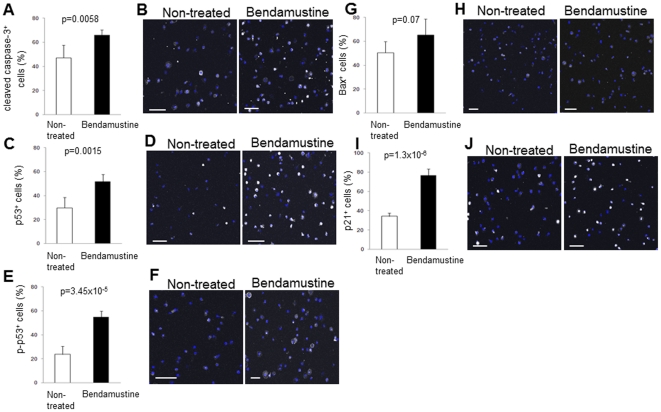
Bendamustine induces p53-dependent apoptosis. Human CD138^+^ mouse CD45^−^ MM cells from non-treated or bendamustine-treated mice were sorted and immunocytochemistry was performed to analyze cleaved caspase-3 (**A, B**), p53 (**C, D**), phosphorylated-p53 (**E, F**), Bax (**G, H**), and p21 (**I, J**). Each group of MM cells was stained with DAPI and anti-fibronectin antibody, anti-p53 antibody, anti-phosphorylated-p53 antibody, anti-Bax antibody, or anti-p21 antibody. At least 100 cells/sample were counted (mean ± SD, n = 10). Bars = 50 µm.

To clarify the molecular mechanism of bendamustine *in vivo*, apoptotic activity was analyzed by detection of cleaved caspase-3. In bendamustine-treated mice, the number of cleaved caspase-3^+^ myeloma cells was significantly increased (Fig. 8A, B). Immunocytochemical analysis of apoptosis-related proteins showed that the levels of total and activated p53, a crucial participant in the apoptotic pathway, were drastically increased in bendamustine-treated myeloma cells (Fig. 8C–F). Although the level of Bax was unchanged by bendamustine treatment (Fig. 8G, H), p21, which induces cell cycle arrest preceding p53-dependent apoptosis, was significantly increased in bendamustine-treated MM cells (Fig. 8I, J).

## Discussion

The present study contributes to the characterization of the MM niche in the BM, which is still incompletely elucidated *in vivo*. Here we show that MM cells preferentially engraft at the metaphyseal region of the BM endosteum and form a complex with osteoblasts and osteoclasts (Fig. 1F, G, 2A). The preferential engraftment of MM cells at the metaphyseal region is thought to be due to the abundance of vasculature in the metaphysis, which is composed of trabecular-rich cancellous bone and a dense vascular network made up of numerous sinusoid capillaries [23]. Similarly, transplanted hematopoietic stem and progenitor cells (HSPC) also engraft preferentially to the metaphysic [24], [25], as do bone metastases of several hematological malignancies [26] and solid tumors [23]. These observations suggest that the vasculature is important for the homing of transplanted HSPC or malignant cells to the bone marrow.

VE-cadherin is a cell adhesion molecule that is crucial for tube formation by endothelial cells [27]. Myeloma cells express various angiogenic factors and promote angiogenesis in the BM. In particular, VEGF [17], its receptors [28], and b-FGF [29] are reportedly produced by MM cells. In this paper, MM cells were also observed to express VE-cadherin, but only *in vivo* (Fig. 3A, B). Since we observed a small population of bendamustine-resistant myeloma cells that were positive for VE-cadherin (Fig. 4E), similar events might occur in the MM *in vivo*. Furthermore, the expression of HIF-2α was increased in VE-cadherin^+^ cells compared to the VE-cadherin^−^ fraction *in vivo* or to *in vitro* cultured MM cells (Fig. 4F). The retention of pimonidazole and the expression level of HIF-1α were also elevated in the VE-cadherin^+^ cell fraction (Fig. 4G, H). These results indicated that HIF-2αspecifically induced MM cells to express VE-cadherin in the BM. This HIF-2α-VE-cadherin pathway is activated in a subset of MM cells in response to hypoxia and might contribute to the maintenance and drug resistance of MM *in vivo*. A recent study has reported that lenalidomide, which is approved for the treatment of MM and myelodysplastic syndromes associated with deletion of the long arm of chromosome 5q, inhibited hypoxia-induced endothelial cell cord formation and HIF-1α accumulation [30]. lenalidomide also inhibited VE-cadherin and another adherens junction protein, β-catenin, in a dose-dependent manner [30]. Combination therapy including anti-angiogenic agents such as lenalidomide may overcome the drug resistance in MM. Recently, the stem cell-like fraction in glioblastoma has been shown to induce a subset of VE-cadherin-expressing endothelial progenitors [31], which form vascular-like structures [32] and maintain tumorigenesis. Thus, the tumor derived or associated vascular cells should be a target of cancer therapy.

During MM progression, in the metaphysical region of the BM, increased numbers of osteoclasts were localized near to, and made contact with, MM cells (Fig. 2B). Because inhibition of osteoclasts reduces angiogenesis and tumor burden in MM [33], both osteoclastogenesis and angiogenesis are considered to progress in parallel to the growth of MM cells. Therefore, these histological findings are in accordance with the current opinion that there is a close relationship between progression of MM, osteoclast formation, and angiogenesis.

ECM proteins play a crucial role in hematopoietic stem cell (HSC) regulation, especially as scaffold proteins [34]. Our observation that ECM structure, especially fibronectin, was increased and disorganized in the non-MM occupied diaphyseal area (Fig. 5B) may indicate migration of MM cells from the metaphysis to the diaphysis. Previous studies have reported that binding MM cells with fibronectin protected MM cells from drug-induced apoptosis [35], [36]. Therefore, targeting not only tumor cells but also the BM niche that sustains tumor growth and survival is an effective treatment strategy for MM.

It is well known that osteoblasts are suppressed by MM cells [37]. We observed disruption of the osteoblast lining on the bone surface in MM mice (Fig. 6A), which suggested a functional decline in osteoblasts, which differentiate from MSCs. It has been reported that MM cells inhibit osteoblastogenesis by blocking the differentiation capacity of mesenchymal and osteoprogenitor cells [38]. According to these previous findings, the increased nestin^+^ mesenchymal cells in the MM BM (Fig. 4G) could be a result of the inhibition of osteoblastogenesis and may have a role in the disease progression of MM. As nestin^+^ mesenchymal cells produce various chemokines and cytokines, the disruption of normal hematopoiesis in MM pathophysiology might be caused by spatial occupancy of tumor growth, disorganized ECM, or reductions in HSC numbers, the latter of which may result from the reduction in osteoblast numbers or from MSC signaling in a paracrine manner.

The present study also clarified the specific molecular effects of bendamustine on MM cells and the MM niche using the NOG-hMM model. Bendamustine reduced VEGF and IL-6 levels in the NOG-hMM model (Fig. 4I). Any direct effects of bendamustine on MM niche cells are not detected at this time, but our results demonstrate that bendamustine could ameliorate MM-induced dysregulation of the BM niche. Future studies will address the direct and indirect effects of bendamustine on MM cells and MM niche cells in the patients and further evaluation of neovascular niches for MM in human samples.

In this study, the mice were not rescued from hind leg paralysis. Nor was the condition of the already diseased mice improved by bendamustine treatment. Bendamustine treatment only delayed the onset of hind leg paralysis (data not shown). The result is possibly due to the remaining MM cells being sufficient to induce clinical symptoms, including hind leg paralysis. In future studies, we will modify our hNOG-MM system by using fewer MM cells for transplantation and a treatment protocol designed to improve MM symptoms.

**Figure 9:**
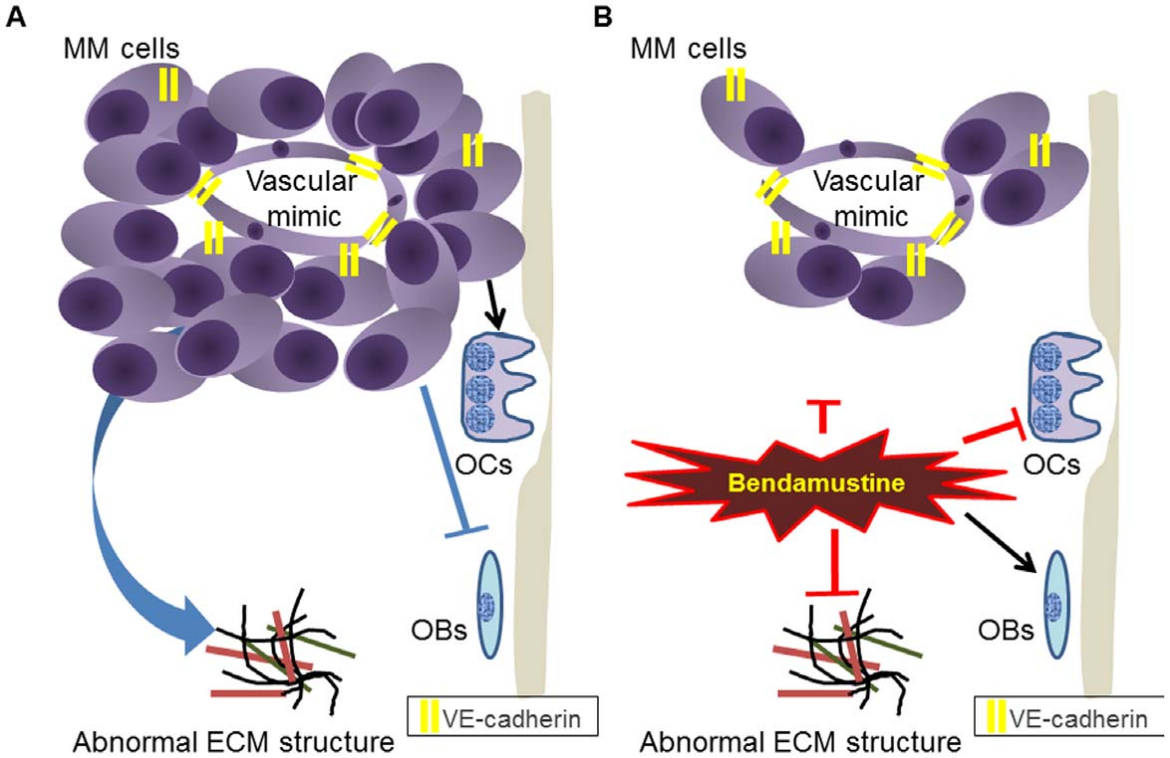
Anti-MM effects of bendamustine on MM cells and the BM niche. MM interacts with several BM microenvironments (**A**). Within the MM BM niche, bendamustine affects both MM cells and the surrounding niche *in vivo* (**B**). OBs, osteoblast cells; OCs, osteoclast cells.

In conclusion, this study characterized the myeloma niche in the NOG-hMM model and examined the effects of bendamustine on both myeloma cells and the surrounding microenvironment *in vivo*. The vascular component of the MM niche plays an important role in supporting MM cells, especially with regard to chemoresistance. Interestingly, these results demonstrated that VE-cadherin^+^ cells are derived from MM cells. Thalidomide [39]; lenalidomide [40]; and bortezomib [41] have all been shown to have anti-angiogenic effects. However, these agents have not been sufficient for the effective treatment of MM. The drug-resistant hypoxic VE-cadherin^+^ HIF-2α^+^ MM fraction identified in this model presents a candidate target for the adjustment of bendamustine dose, combination therapy, or selective targeting (Fig. 9). The NOG-hMM model described here is a useful system for the analysis of the *in vivo* dynamics and kinetics of myeloma cell pathophysiology and for observing the interactions of MM cells with other components of the microenvironment, as well as for the development of novel anti-MM therapies.

## Materials and Methods

### Mice

NOG (NOD/SCID/γ_c_^null^) mice [12] were purchased from the Central Institute for Experimental Animals. All animal experiments were approved by the Keio University School of Medicine's ethical review board (approval ID: 08104).

### Reagents and antibodies

Bendamustine was kindly provided by SymBio Pharmaceuticals Ltd. The following antibodies (Abs) labeled with fluorophores were used for flow cytometric analysis and cell sorting: anti-mouse CD45 (30-F11; BD Biosciences), anti-human CD138 (BD Biosciences), and anti-human VE-cadherin (16B1; eBioscience). For immunohistochemical staining of BM sections or immunocytochemical staining of MM cells, the following Abs were used: mouse anti-CD138 (AbD seroTec), rabbit anti-ALP (R&D), anti-MMP9 (Chemicon), anti-GFP (MBL), rabbit anti-fibronectin (Dako), rabbit anti-type IV collagen (AbD seroTec), rabbit anti-α-laminin (Sigma), rat anti-nestin (BioAcademia), FITC-conjugated anti-pimonidazole (Chemicon), rabbit anti-HIF-1α (Santa Cruz), and rabbit anti-HIF-2α (Santa Cruz).

### Quantitative RT-PCR

Quantitative PCR was performed as described previously [42]. Total RNA was prepared with the RNeasy Mini Kit (Qiagen) and was reverse-transcribed with Superscript VILO (Invitrogen) to prepare cDNA. Diluted cDNA samples were analyzed with SYBR Premix Ex Taq II (TaKaRa) using readymade primer sets for each gene (TaKaRa), according to the manufacturer's instructions. Primer sets were used for human IL-6 (HA032507), VEGFA (HA110047), FGF-2 (HA035991), or β-actin (HA031582), which served as an endogenous control. Data were analyzed using 7500 Fast System SDS software, version 1.3.1.

### Human MM model

The human myeloma cell line, U266, was purchased from the American Tissue Culture Collection (Manassas, VA [13]; and was stably tagged with EGFP with a lentiviral vector. 2×10^6^ U266-EGFP cells were intravenously inoculated into irradiated NOG mice (2.4 Gy). After transplantation, mice were given sterile water containing hydrochloric acid and G418 disulfate in aqueous solution (Nacalai Tesque).

### Flow cytometry

Stained cells were analyzed and sorted on a SORP FACS Aria (BD Biosciences).

### Immunohistochemistry

Femurs were fixed in 4% paraformaldehyde, decalcified in EDTA, and embedded in OCT. Frozen sections were cut using a cryostat (MICROME, model HM550). Slides were washed three times in PBS and stained for TRAP activity (Primary Cell Co., Ltd.). For immunohistochemistry, femoral frozen sections were washed three times in PBS, blocked with a protein blocker (DAKO), and then incubated with primary antibodies overnight in humidified chambers at 4°C. The sections were then washed three times in PBS and reacted with fluorophore-labeled secondary antibodies and a nuclear stain (TOTO3 or DAPI; Molecular Probes). Fluorescence images were obtained using a confocal laser-scanning microscope (FV1000; Olympus) in the linear range to avoid fluorescence saturation. Scanning was performed in the sequential laser emission mode to avoid cross-talk between the fluorescence channels. For detection of cellular hypoxia, six weeks after MM cell transplantation, NOG mice were injected with 60 mg/kg pimonidazole (Pimo; Chemicon) 90 min before sacrifice. Human CD138-gated VE-cadherin–positive or –negative fractions were sorted for immunocytochemistry.

### Histological evaluation

Thoracic vertebra were fixed in 4% PFA and decalcified in EDTA, followed by paraffin embedding and sectioning. Morphological evaluation of the sections was performed following hematoxylin and eosin (HE) staining, reticulin staining, and Masson trichrome staining.

### Human IgE measurement

Serum levels of human IgE in NOG recipients was measured using a human IgE ELISA kit (Phadia, SW) following the manufacturer's instructions.

### Statistical analysis

*P*-values were calculated using two-tailed unpaired Student's t-tests (for experiments with normal distribution), Wilcoxon tests (for two-group experiments with non-normal distribution), or Tukey's multiple comparison tests (for multiple-group experiments).

## Supporting Information

Table S1 **U266-GFP cells rarely fused with mouse endothelial cells.** Fluorescence in situ hybridization (FISH) analysis of VE-cadherin positive and VE-cadherin negative cell fractions, and cultured U266-GFP cells. Each fusion rate is represented.
(PDF)
