# Supplementary material for: Neovascular Niche for Human Myeloma Cells in Immunodeficient Mouse Bone
Source: PLoS One. 2012 Feb 7;7(2):e30557. doi: 10.1371/journal.pone.0030557 (PMC3274528; doi:10.1371/journal.pone.0030557)
Supplement: Table S1 — U266-GFP cells rarely fused with mouse endothelial cells. Fluorescence in situ hybridization (FISH) analysis of VE-cadherin positive and VE-cadherin negative cell fractions, and cultured U266-GFP cells. Each fusion rate is represented. (PDF) [file pone.0030557.s001.pdf]

Table S1

	U266-GFP cells	VE-cad (-) $CD138^+$ cells	VE-cad (+) $CD138^+$ cells
Fusion rate (%)	0/1014 (0)	11/23000 (0.05)	10/14700 (0.07)
